# Synthesis of a novel antiweathering nanocomposite superhydrophobic room temperature vulcanized (RTV) silicon rubber enhanced with nanosilica for coating high voltage insulators

**DOI:** 10.55730/1300-0527.3361

**Published:** 2021-12-27

**Authors:** Khalid K. ABBAS, Mayyadah S. ABED, Ali F. JASIM

**Affiliations:** 1 Department of Materials Engineering, University of Technology, Baghdad, IRAQ; 2 Ministry of Electricity General Directorate of Electric Energy Transportation for Euphrates Middle Region, Baghdad, IRAQ

**Keywords:** Nanosilica, silicon rubber, heterojunction superhydrophobic, high voltage, glass insulator, heterojunction nanocomposite

## Abstract

A new nanocomposite superhydrophobic of the RTV (room temperature vulcanized) silicon rubber reinforced with a different percentage of nanosilica was prepared by a two-stage sol-gel route to obtain a superhydrophobic surface coating on high voltage glass insulator, preventing the dust-water droplet from adhering to its surface. The cold spraying technique was utilized to build up a thin nanocomposite superhydrophobic layer on the glass insulator containing different percentages of the nanosilica particles, such as 23 wt %, 33 wt %, and 44 wt % with RTV silicon substrate. The synthesized nanocomposite was analyzed using the contact angle, roughness, adhesion, hardness, and dielectric strength tests. Moreover, the prepared RTV silicon rubber/nanosilica superhydrophobic nanocomposite layer was characterized using the field emission scanning electron microscope (FESEM), X-ray diffraction (XRD), Fourier transform infrared spectroscopy (FTIR), and the particle size analysis test. Based on the results, the nanosilica particles were well-incorporated into the RTV silicon rubber, obtaining an excellent homogenous distribution thin layer on its surface, supporting its capability to be a superior superhydrophobic surface. The results reveal that the RTV silicon rubber/33wt % nanosilica was the best as a superhydrophobic behavior with a contact angle reaching higher than 158° ± 3; also, a significant change in the dielectric strength was obtained to be 25.5 kV (using a speed voltage of 5.0 kV/s). Importantly, the flashover test was also conducted, and it was found that there was a significant change in the leak current between the coated and uncoated samples. The leak current of the coated sample with a superhydrophobic nanocomposite was reduced to 2.5 mA, while the uncoated sample became 3.2 mA using a voltage load value of 60 kV. The results presented here may improve the nanocomposite material as an antiweathering superhydrophobic thin layer supported by the prepared nano-SiO_2_ particles against the dust-water droplets which may be adhesive to the high voltage glass insulator.

## 1. Introduction

Bad weather can collect a lot of dust, contaminating the high voltage glass insulator, which is a significant issue in the electricity distribution field due to the high cost of the cleaning process [1,2]. For that reason, academic and industrial researchers are interested in the coating technique for obtaining new superhydrophilic materials. Glass coating with a superhydrophobic nanocomposite material has a wide range of applications due to it is unique characteristics, such as a self-cleaning technique with low adhesion surface [3,4], corrosion resistance [5,6], antiicing [7,8] and excellent antibacterial properties [9,10]. To achieve a self-cleaning glass insulator, a coating of superhydrophobic nanocomposite materials can be applied, which has the ability to reduce not only the dust accumulation but also the complex rain salts that accumulate on its surface. The superhydrophobic nanocomposite material can be defined as a unique material that is extremely difficult to wet with a contact angle of over 150^o^.

Many researchers have been investigating the coating techniques of the high voltage glass insulator via the spin coating [1], the deposition coating [11], the sol–gel method [12], and the laser etching technique [13]. However, these techniques have low efficiencies, and the flashover can occur after a while, even using composite materials. Coating glass with new transparent superhydrophobic materials has attracted a lot of attention in recent years due to its unique properties, such as low viscosity, excellent flow-ability, high temperature resistance, and ageing resistance. Additionally, roughness and low surface energy are important factors influencing the coating technique of the superhydrophobic materials. Furthermore, the high roughness is mainly attributed to the inhomogeneity of the coating surface, which may hinder the optical clarity and then reduce the efficiency of the high-voltage insulator by increasing the flashover process. In contrast, the surface’s transparency is an inherent characteristic of the high voltage glass insulator, which plays a vital role in the light scattering on its surface. Therefore, transparent superhydrophobic nanocomposite materials are preferred when coating the surface of the high-voltage glass insulator.

Numerous studies have attempted to protect the glass surfaces against encrustation, corrosion, icing, and fouling using coatings containing polymer films, hydrophobic solid fillers, and hydrophobic liquids. One disadvantage to using such materials is that achieving multipurpose protection is not versatile enough to protect the glass insulator against damage caused by different circumstances. Therefore, a new nanocomposite superhydrophobic material has become an urgent demand in the industrial field. Previous research has concluded that a hybrid coating with micro/nanoscale is required to minimize the optical loss while maintaining the superhydrophobic effectiveness [2]. For example, coating superhydrophobic surfaces on tetra needles like zinc oxide whiskers (T-ZnO)/silicon rubber (SR) and ordinary zinc oxide (O-ZnO)/SR composites were developed using a simple powder scattering technology. The static contact angle (CA) between water and the surface of T-ZnO/SR was 154.26 ±1.2°, and the rolling angle (RA) was 51.8°. The static CA between water and the O-ZnO/SR surface, on the other hand, was 150.47 ±1.1°, and the RA is higher than 90°. The T-ZnO/superhydrophobic SR’s surface has such reduced water adhesiveness and a superior self-cleaning capability [14]. In addition, a transparent superhydrophobic surface with a dual-scale roughness on the nanoscale aminopropyltriethoxysilane (APTS)-functionalized silica nanoparticles onto various substrates was synthesized using heptadecafluoro-1,1,2,2-tetrahydrodecyl trichlorosilane with a contact angle of more than 160^o^. However, the prepared nanoparticles are dip-coated onto the silicon wafer, which has no influence on water contact angle and has less cleaning ability. But, when the substrate was dip-coated with nanoscale particles, a hydrophobic surface was successfully formed, and the contact angle degree depended on the particle size and the dip-coating concentration [15].

Adding silica nanoparticles to the electrospinnable polymer solution is another way to generate superhydrophobic fibrous polystyrene surfaces. With a silica content of 14.3 wt % in the PS fibers, the inorganic nanoparticles associated with the DMF (Dimethylformamide), which increased the roughness of the fiber surface, thus, modulated the superhydrophobic properties: the highest water contact angle (CA = 157.2°) was obtained. For instance, fluoroalkyl end-functionalized polystyrene additives could be utilized to convert the morphology of electrospun fibers [16]. A simple and inexpensive process for producing a superhydrophobic coating has been developed using polypropylene (a basic polymer) and proper mixing solvents by heating to manage the surface roughness of the sample. The water contact angle of the resulting gel-like porous coating was 160°. As long as the solvent mixture does not dissolve the underlying substrate, the procedure can be used on a variety of surfaces [17].

Another example is injecting lubricant oil into a porous tube-like SiO_2_ composite, producing a very transparent, durable, slippery coating. It was discovered that SLIPS (slippery lubricant-infused porous surfaces) had an ultra-low ice adhesion strength surface that reached 17 kPa and a capillary action inside the unique structure with self-healing ability [18]. However, this method is not capable of cleaning up the dirty droplets on the glass insulator. For example, Siria and coworkers demonstrated that the deposited single layer of dual-scale silica particles with high roughness can be caused by applying a hot-steam deposition of an alkylsilane mixture in open air yielding a well-controlled thickness in the range of 200–400 nm with a contact angle of 173^o^. It showed outstanding superhydrophobic characteristics, resulting in high stability of droplet compression, water jet erosion, and UV irradiation with induced deterioration. This indicates that they are suitable for outdoor use [2], but the single layer of the silica particles was reduced after a while. In a new investigation, Zhu examined the superhydrophobic coatings on the high voltage glass insulator, improving its performance by using PDMS (Polydimethylsiloxane) with nano particles of SiO_2_ obtaining a superhydrophobic surface with adjustable adhesion and a great self-cleaning property, as well as good durability and thermal stability, reaching a contact angle of more than 155.13° [19].

One of the critical characteristics that the superhydrophobic coating can significantly influence is the adhesion of pollutant dust to the glass insulator. The dust-water droplets adhere to the high voltage glass insulator, resulting in a flashover issue. As a result, preventing these droplets from adhering to the glass insulator is a critical issue. These droplets can be manipulated by increasing the contact angle using a novel superhydrophobic nanocomposite material with a tunable adhesion ability [20]. The measurement of the advanced contact angle (CA) is a significant matter, which corresponds to the maximal solid/liquid surface tension and the minimal work adhesion calculated according to the Dupre equation (Eq.1) [21].


(1) $$W_{min}=\gamma \;(1+\text{cos }\theta )$$

where, *W_min_* is minimal work, *γ* is the surface tenstion, while cosΘ is the advance contact angle.

It is well known that a hydrophobic solid’s roughness increases with its hydrophobicity [22]. The contact angles were between 100 and 120° with flat objects, but they can reach up to 160–175° when the surface has more roughness or micro-textured structure [23].

One of the chemical materials that is considered to have good surface adhesion is RTV silicon rubber. The RTV silicones are made from mixtures of silicone polymers, fillers, and organo-reactive silane catalysts. Silicones are formed from the Si-O bond, but they can have a wide variety of side chains [24]. Recently, there has been a lot of interest in using RTV silicon rubber in the industrial applications, such as aviation, aerospace, consumer electronics, and microelectronics [25]. The electrical properties of RTV silicon rubber depend on temperature and are strongly affected by exposure to water. Accordingly, the resistivity of 10^16^ Ω cm under dry conditions at 20 °C decreases to 10^12^ Ω cm at 160 °C with 50% R.H. (relative humidity) [24]. Therefore, new nanoparticles incorporated into RTV silicon rubber could improve the resistivity and resistance under the environmental temperatures.

The spin coating approach was used to synthesize optical-transparent superhydrophobic TEOS-silica composite films in a one-pot single step. Silica sols were synthesized by maintaining a constant molar ratio of TEOS/CH_3_OH/H_2_O (0.1 M NH_4_F) of 1:33.15:6.06. The transparency of the nanocomposite films was reduced from 93 to 84% by increasing the silica concentration of the coating solution. The contact angle of transparent surfaces was found to be 170°. Under ambient circumstances, the surface stability remained essentially constant for several months [26]. Ecosystem contamination can reduce the reliability of electrical transmission systems and lead to flashovers and arcing over the entire glass insulator. Due to its self-cleaning effect, a superhydrophobic coating with a nanomaterial would solve this problem by reducing the dirt-particles’ adhesiveness onto the surfaces of the glass insulators. A previous study claimed that the superhydrophobic RTV silicone rubber was used as a coating material on a glass substrate, obtaining a contact angle of more than 145° with good UV durability. This study utilized a superhydrophobic RTV coating, which is made from the mixing of a hydrophobic RTV-1 liquid silicone rubber with fluoric nano particles, alumina tri-hydrate (ATH), and a solvent [27]. However, the prepared material still has a low contact angle, making it difficult to apply in a variety of industrial situations.

The goal of this research is to synthesize a novel superhydrophobic nanocomposite material using the RTV silicon rubber with different ratios of the prepared nanosilica particles (SiO_2_) to reduce the flashover dilemma in high voltage glass insulators. To the best of the authors’ knowledge, no report has been found so far using the synthesized nano-silica particles as a nanocomposite material incorporated with RTV silicon rubber, enhancing the superhydrophobicity. The characterizations of the prepared RTV silicon rubber/nanosilica were investigated using XRD, FESEM, FTIR, and particle size analysis. The prepared nanomaterial thin layer was employed in different real regions to be assured that it has good durability with no flashover behavior that can be obtained during its applications.

## 2. Experimental work

### 2.1 Materials

**Scheme 1:**
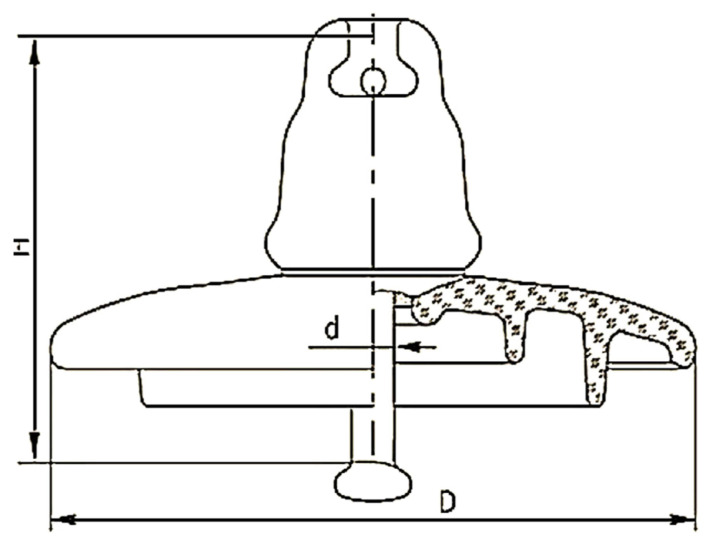
Schematic illustration of the high voltage glass insulator shows the dimensions, performance characteristics, and components.

Tetraethyl orthosilicate (TEOS, 98%) and toluene (purity, 99.5%) were acquired by (Sigma–Aldrich Comp., USA). The RTV silicon rubber with the commercial name EL1037 T was purchased from (Sunny Zhou Company, China). The base polymers are polydimethylsiloxanes with terminal hydroxyl groups with a viscosity of 150 Pa.s. The N-Hexane was obtained from (Alpha Chemika, India, 97%), while the ethanol was purchased from (Pronto Chemical CO. Turkey, 96%). The 2-propanol was acquired from (Rubilabor Chemical, Spain, 99.5%), and the nitric acid (HNO_3_, 70 %) was supplied by Alpha Chemika, India Company. As illustrated in Scheme 1, the high voltage glass insulator was supplied by the North Karbala - Al-Ukhaidir station company (Iraq). All the chemical reagents were utilized as laboratory grade and were obtained with no further purification. The mixture solutions were prepared using deionized water (DI).

### 2.2. Preparation of the nanosilica

The silica nanoparticles were synthesized using the sol-gel method with slight modification. Briefly, 2 mL of TEOS as a precursor was mixed with 5 mL of ethanol solvent in a conical flask container and magnetically stirred at room temperature for 30 min. Then, 2.7 mL of HNO_3_ was added gently as droplets to this mixture for 120 min, raising the temperature up to 60 °C with continuous stirring until a homogenous gel-solution was obtained. After that, the sample was collected and dried at 100 °C for 24 h. Finally, the collected sample was sintered in a muffle furnace at 600 °C for 4 h to obtain fine, fluffy nanoparticles [19].

### 2.3 Synthesis of the RTV silicon rubber/Nanosilica particles

To obtain a heterogeneous nanocomposite material, the RTV silicon rubber (1 g) was added to 50 mL of toluene with magnetic mixing at 60 °C for 30 min until a homogenous solution was reached. After that, different weight percentages of the prepared nanosilica, such as 23 wt%, 33 wt%, and 44 wt% were added to the homogenous solution. To reach an ultrahigh homogenous solution of 100 mL of ethanol, different concentrations of nano-silica were prepared at 5 wt%, 10 wt%, 15 wt%, 20 wt%, 30 wt%, 40 wt% and 50 wt%. When this solution mixture was mixed with RTV silicon rubber, it became extremely saturated with nano silica and then precipitated, resulting in a nonhomogenous solution; however, at low concentrations, the resultant mixture was also nonhomogenous, resulting in a nonsmooth surface. Therefore, the perfect concentrations were selected, such as the ones including nanosilica particles in the rates of 23 wt%, 33 wt%, and 44 wt%. In more details, the synthesized nanosilica was placed in 100 mL of ethanol, and then the used flask was put through ultrasonication process (400 Watts, 60 Hz, 1/1 pulses) for 30 min to achieve an ultrahigh homogenous solution. Then, the obtained solution was magnetically mixed with the RTV silicon rubber solution at room temperature for 10 min to acquire a new nanocomposite of the RTV silicon rubber incorporated nanosilica particles. Finally, the RTV silicon rubber/nanosilica particles were filtrated and dried at 60 °C for 5 h, and then they were collected to be ready to be used as a superhydrophobic coating material.

### 2.4 Coating the high voltage glass insulator technique

A cold spray technique (INGCO SPRAY GUN 20V SOLO, China) was applied in this study with a slide angle of up to 45°. The spray distance was 50 cm from the sample, and the spraying pressure was in the range of 1.5–2.0 bar. Each sample was sprayed three times to achieve a good layer thickness. Then, the coated glass insulator was dried in an oven at 100–120 °C for 2 h. After the solvent evaporation, the nanosilica particles were immobilized on the RTV silicon rubber at approximately 23 wt%, 33 wt%, and 44.5 wt%.

### 2.5. Characterization

The crystal structure was characterized by the X-ray diffractometer model XRD 6000/Shimadzu/Japan. The work condition was (X-ray Tube: Cu (1.54060 A) voltage: 40.0 kv, current: 30.0 ma, scan range: 10–90, step size: 0.2, count time: 1.2 seconds). The surface morphology of the synthesized samples was observed by the field emission scanning electron microscope (FESEM), Zeiss, Germany, (FEI, model Inspect F50, USA) at an acceleration voltage of 5 kV with a magnification of 30 and 60 kX. The nonorganic materials groups were evaluated using Fourier transformed infrared (FTIR) spectroscopy (Bruker, wavelength in range of 400cm^−1^–3500cm^−1^). To determine the particle size distribution and surface tension stability, the particle size analyzer and zeta potential (HORIBA, S-Z 100 Japan) were also used. The particle size measurement was done at these conditions: 90° scattering angle, and the holder temperature was 25.2 °C with a pressure of 0.892 mPa s dispersed at a medium viscosity. Whereas the measurement of the zeta potential was studied at conditions of 0.894 mPa s dispersed in a medium viscosity with a 0.054 mS/cm conductivity and a 3.9 V electrode voltage.

### 2.6 Coating efficiency analysis

The wettability of the samples was determined using a contact angle measurement device (Crating Nano Technologies Inc., Model CAM120, Taiwan) by dropping the deionized water on the coated surface at a temperature of 25 °C. More tests have been done using the digital screen with a monitoring program measuring the contact angle. The sample’s roughness was also investigated by a handheld surface roughness tester with a graphical display (TR200 Salu Tron). Five roughness measurements were taken in different regions of the sample to obtain an average roughness value. The dielectric strength of the specimens was measured using a high voltage supplier with a range of (0–60 kV) and a frequency of 50 Hz, model PAUR-PGO-S-3 from Germany. In this test, the total breakdown voltage is determined by placing electrodes on opposite surfaces of a specimen disc and then increasing the potential difference between the electrodes until the material can no longer resist the flow of the current. The test specimen size was in the shape of a 40 mm diameter sphere with a standard thickness of 2 mm. To be ready for measurement at room temperature, insulation oil was used as an embedding medium. The imposed voltage was 50 Hz AC with an increasing rate of 2 kV/s. A flashover is one of the most important tests that should be performed to prevent a power outage. The setup of this test is shown in the supplementary material S1 (BAUR-PGK HB). Water was mixed with a pollutant substance similar to the volatile substances that are available in the atmosphere, such as flying dust. The applied voltages were in the range of 1–60 kV.

## 3. Results and discussion

### 3.1. X-ray diffraction

**Figure 1:**
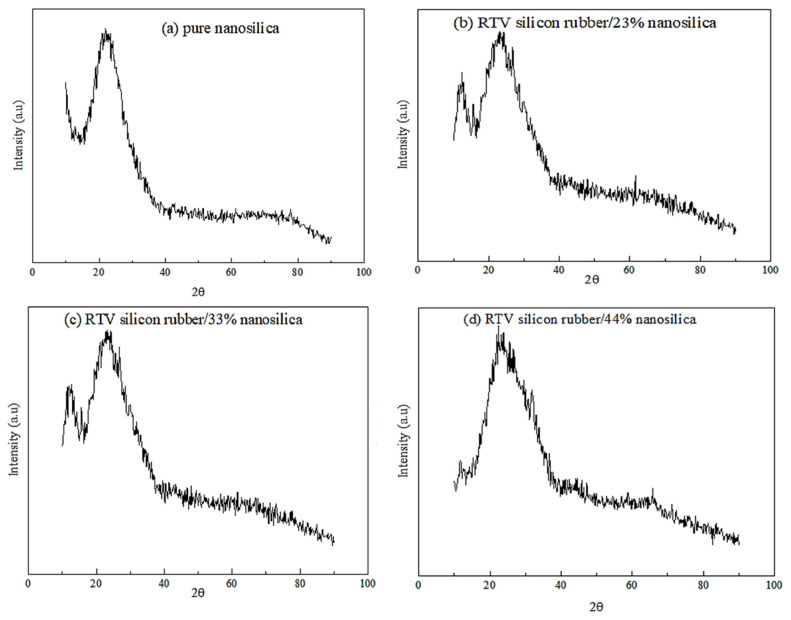
The X-ray diffraction spectra of the prepared nanosilica, (a) pure nanosilica (b) RTV Silicon rubber/23 wt% nanosilica, (c) RTV Silicon rubber/33 wt% nanosilica & RTV (d) Silicon rubber/44 wt% nanosilica.

The XRD pattern signals were investigated and illustrated in Figure 1. Different nanocomposite materials were studied, including the prepared nanosilica particles, RTV silicon rubber/23 wt % nanosilica, RTV Silicon rubber/33 wt% nanosilica, and RTV silicon rubber/44.5 wt% nanosilica. The XRD pattern of the nano-SiO_2_ particle, as shown in Figure 1a, is primarily composed of amorphous silica with low crystalline [28]. The diffractogram of the nano-SiO_2_ is also noticed at a peak of 2θ = 22°, which confirms the amorphous nature of the silica nanoparticles. Furthermore, the XRD pattern reveals the absence of any ordered crystalline structure, indicating a disordered structure, due to the silica appearing as an amorphous particle [29]. The grain crystallite size was calculated to be approximately 6.22 nm using Scherer’s equation (Eq. 2):


(2) D=Kλ/(β cos θ)

Where D is the mean size of crystallites (nm), K is the crystallite shape factor with a good approximation of 0.9, λ is the X-ray wavelength, B is the full width at half the maximum (FWHM) of the X-ray diffraction peak in radians unit, and θ is the Braggs’ angle (deg.) Interestingly, this result indicates that the preparation process of the nanosilica was successfully carried out. According to Figure 1b, Figure 1c, the XRD patterns of the nanocomposites of RTV silicon rubber after adding 23 wt% nanosilica and RTV silicon rubber after adding 33 wt% nanosilica show no shifting peaks, but a new intense peak was observed at 2Θ = 11.56° attributable to the diffraction peak of the RTV-silicon rubber, indicating that there exists a certain degree of ordered structure that is usually accompanied by an internal partial regularity of the RTV [30]. Also, this confirms that the RTV silicon rubber has interacted with the nanosilica particles with slight crystallinity. In Figure 1d, the intense peak at 2Θ = 11.56° almost disappeared when the nanosilica particles were increased to 44 wt%, indicating that the disordered structure of the nanosilica was affected by the structural RTV silicon rubber. Therefore, any increase in the nanosilica particles can destroy the structure of the RTV silicon rubber.

### 3.2. Field emission scanning electron microscope (FESEM)

**Figure 2:**
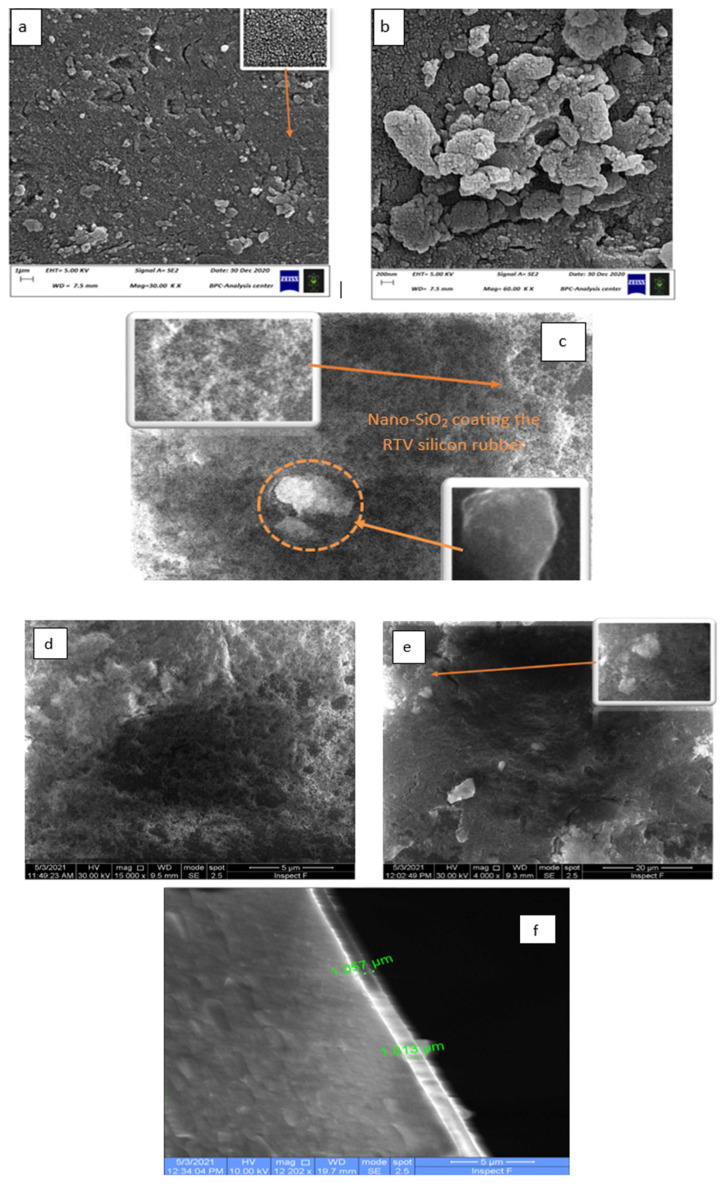
The FESEM images. a,b) SEM images of the prepared nanosilica. c,d) FESEM images of the coated sample with 23 wt % nano-SiO_2_/RTV silicon rubber. e) FESEM images of the coated sample with 33 wt % nano-SiO_2_/RTV silicon rubber. (f) The cross section thickness of the coating material (RTV rubber/nano-silica) on to the glass substrate.

High magnification photos were obtained using FESEM analysis that displayed the microstructure of the prepared nanosilica. The RTV silicon rubber incorporated different concentrations of nanosilica, such as 23 wt%, 33 wt%, and 44 wt %, were studied. Figures 2a and 2b show the nanosilica in the shape of a nano-sphere morphology. The clusters were formed from nanosilica with no cavities, which reduced the pore size and resulted in high agglomeration behavior. This behavior can be helped by completely covering the glass substrate. Figure 2c illustrates a homogenous distribution of the nanosilica within the silicon rubber matrix at concentrations of 33 wt%, which seems to have a colloid, cotton-net-like structure, with a well-distribution of the nano-SiO_2_ particles, confirming intermolecular dispersive interaction between RTV silicone rubber and nanosilica particles. Meanwhile, 44 wt% and 23 wt% of the nanosilica were segregated significantly into the polymer matrix of the RTV silicon rubber with high grooves and cavities that can be easily noticed in Figures 2d and 2e, respectively. This phenomenon is due to a high viscosity with disordered distribution of particles resulting when the incorporated nano-SiO_2_ is 44 wt% and 23 wt% in the coating matrix, which leads to an impede continuous coating layer building up on the glass substrate. These findings are consistent with those of a previous study [22]. Thus, increasing the nanosilica particles in the nanocomposite RTV silicon rubber leads to a deficiency in the cold coating process. The cross-section coating thickness of the RTV silicon rubber/nanosilica was also investigated in Figure 2f. It can be noticed that a homogenous thickness layer along with the glass substrate surface was obtained as an average thickness of 1 μm.

### 3.3. FTIR analysis

**Figure 3:**
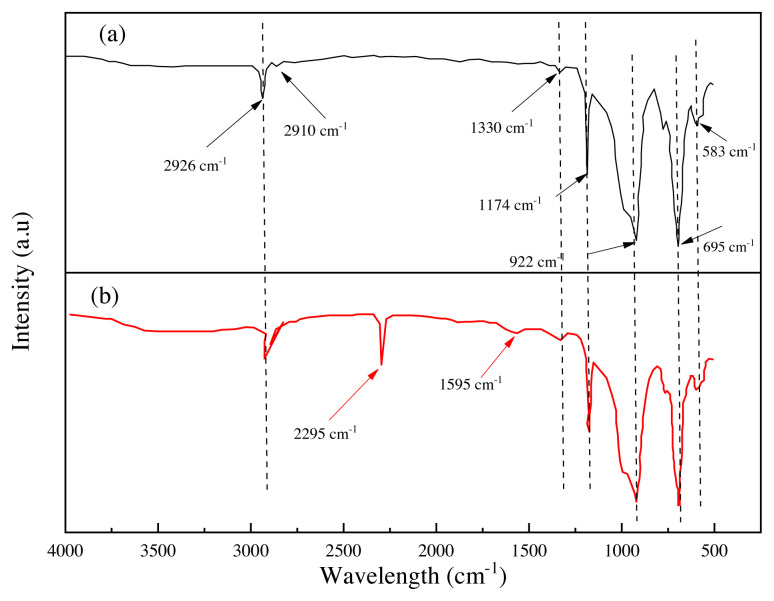
(a) The FTIR analysis of the RTV silicon rubber, (b) the FTIR analysis of the nanocomposite of the RTV silicon rubber/33 wt% nano-SiO_2_.

**Scheme 2:**
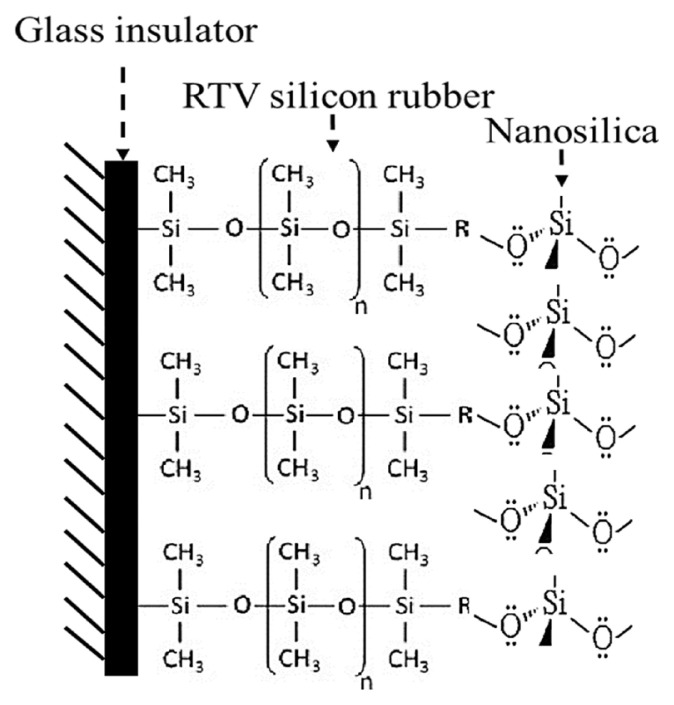
Schematic illustration of the bond interaction network between the RTV silicon rubber and the nano-SiO_2_.

The Fourier-transformed infrared spectroscopy analysis was performed, including the pristine RTV silicon rubber and RTV silicon rubber/33 wt% nano-SiO_2_ as illustrated in Figures 3a, 3b. From Figure 3a, the spectrum signals of the pristine RTV silicon rubber at 2926 cm^−1^ and 2910 cm^−1^ are ascribed to the stretching vibration absorption of the C-H in the -CH_3_ bond. The peaks at 1174 cm^−1^ and 922 cm^−1^ are assigned to the scissoring vibration absorption of Si-CH_3_ [31]. The sharp intensity peak at 695 cm^−1^ and the small signal peak at 583 cm^−1^ are attributed to the stretching vibration absorption of Si-O-Si [32]. Additionally, a small spectra peak at 1330 cm^−1^ indicates the stretching vibration of the H-bonded silanol group Si-OH [33]. When compared to the curve in Figure 3b, asymmetric peaks appeared, and an intense absorption sharp peak of the bonded –Si (nano-SiO_2_) with –OCH_3_ (RTV silicon rubber) was observed at 2295 cm^−1^, confirming an interaction between the nano-SiO_2_ particles and the structure of the RTV silicon rubber. Furthermore, a signal peak was observed at 1595 cm^−1^ assigned to a group of -CH_3_-Si-CH_3_-. These characteristics suggest that nano-SiO_2_ particles successfully interfered with RTV silicon rubber morphology, obtaining a new network of O-CH_3_-Si-CH_3_-O. Scheme 2 describes the dispersive intermolecular interactions between RTV silicone rubber and the nanosilica particles network. Therefore, the high incorporation of the nanosilica particles within the RTV silicon rubber was successfully obtained.

### 3.2. Particle size and zeta potential measurement

**Figure 4:**
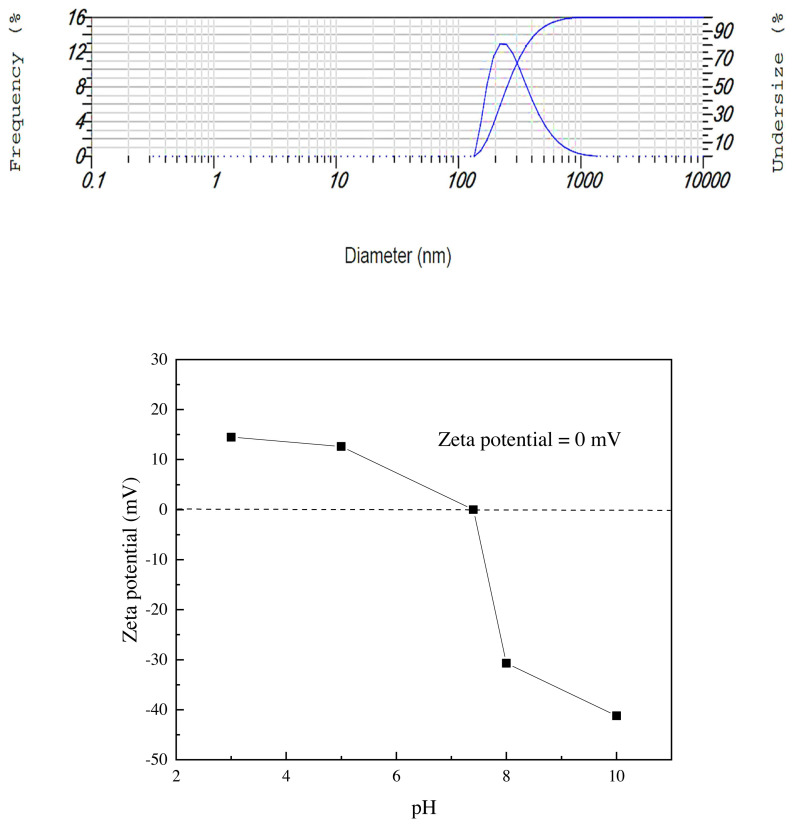
Nanosilica Particles size distribution onto the RTV silicon rubber. The Conditions: the scattering angle 90°, the temperature of the holder is 25.2 °C; the dispersion medium viscosity is 0.892 mPa.s.

The particle size analysis of the prepared nanosilica was illustrated in Figure 4. Interestingly, this result confirmed that the nano-SiO_2_ particles were detected with a high dispersion on the glass substrate. The nanosilica particle size reflects excellent adhesion of the nanoparticles onto the glass substrate, with identical topography being obtained.

**Figure 5:**
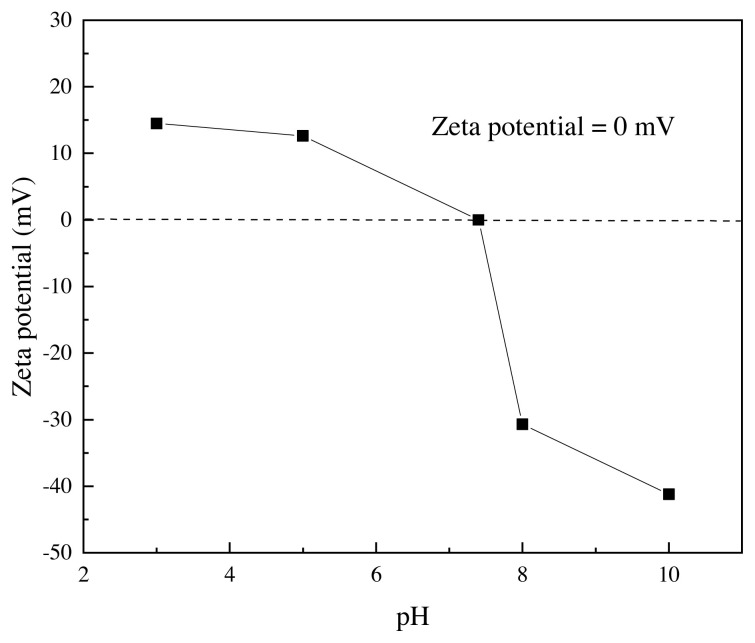
The zeta potential curve using a solution of ethanol/water (40%/60%) for dispersion the nanoparticles of the SiO_2_. Conditions: Temperature of the holder is 25.0 °C, dispersion medium viscosity is 0.894 mPa.s, Conductivity is 0.054 mS/cm and the electro voltage is 3.9 V.

The zeta potential analysis determines the stability behavior of the collided solution. It is well understood that a high absolute value of the zeta potential indicates a more stable state of colloidal systems, and that potential levels greater than +30 mV or less than −30 mV allow for an essentially stable suspension [34]. The nanosilica particle solution was prepared for the zeta potential test using an ethanol: water system (40:60) with an adjusted pH solution of 0.1 M NaOH or 0.1 M HCl. As displayed in Figure 5, it can be clearly noticed that the stability of the particles in the colloid solution of the RTV silicon rubber when the Zeta Potential value increases from 0 to −41.2 mV at pH = 7.4 and 10.0, respectively. This behavior indicates a stable colloid system, which causes good nanosilica particle dispersion during the coating system with no agglomeration. Additionally, a very low electrophoretic mobility was obtained to be −0.000319 cm^2^/Vs. As a result, the prepared nanosilica in a wide range of zeta potential values can be successfully applied with good colloid dispersion; the cold spraying coating technique significantly improves the liquid suspension stability with low surface tension behavior [35]. Thus, the nanosilica particles have excellent stability on the glass substrate surface, even when high humidity is present in the environment.

## 4. Coating properties

### 4.1. Wettability and adhesion

**Figure 6:**
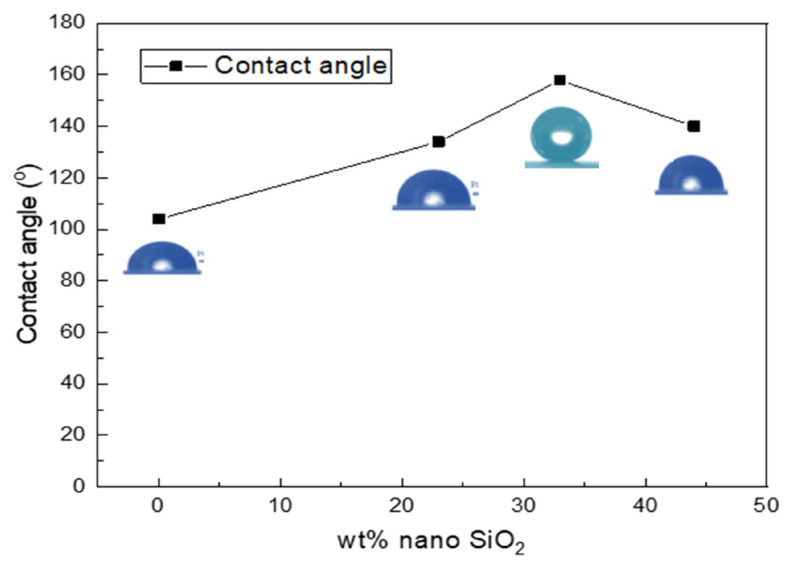
The contact angle onto the glass substrate using different concentrations of the nanosilica such as 23, 33, 44 wt% nano-SiO_2_.

**Figure 7:**
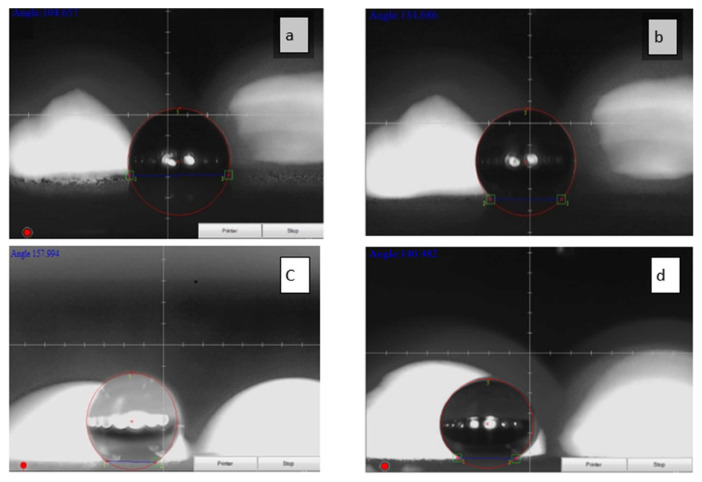
a) The RTV silicon rubber with 0 % Nano-SiO_2_. b) The RTV silicon rubber/23 wt% nano-SiO_2_. c) The RTV silicon rubber/33 wt% nano-SiO_2_. d) The RTV silicon rubber/44 wt% nano-SiO_2_.

The measurement of the contact angle was performed using different concentrations of the nanosilica particles immobilized on the RTV silicon rubber. The 5 μL water-droplet was used and maintained as a spherical shape on the sample surface. As illustrated in Figure 6, the contact angle values using 0, 23, 33, and 44 wt% nano-SiO_2_ particles incorporated onto the RTV rubber matrix are 104°±5, 134°±3, 158°±3, 140°±5, respectively. To discuss these results, the RTV silicon rubber with no nanosilica content shows good hydrophobic behavior with a smooth surface on the glass substrate, reaching a contact angle of 104°±5 as illustrated in Figure 7a, but it still requires more improvement due to the fact that this contact angle is not suitable to be a superhydrophobic phenomenon. The hydrophobicity can be increased gradually when adding 23 wt% of nanosilica to the nanocomposite RTV silicon rubber, obtaining a contact angle of up to 134°±3 as displayed in Figure 7b. Interestingly, the superhydrophobic behavior occurred at a water contact angle of 158° ± 3 when adding 33 wt % of nanosilica to the nanocomposite RTV silicon rubber, indicating that the superhydrophopicity increased with well-distribution of the nanosilica particles as a thin contact strips, obtaining a good roughness zone as displayed in Figure 7c; however, the contact angle was deteriorated to 140° ± 5 when adding more nanosilica particle to the RTV silicon rubber nanocomposite reaching to 44 wt % due to the fact that a high conglomerates is noticed with less adhesion droplets on its surface as shown in Figure 7d. That means the optimum superhydrophobic coating occurred at 33 wt % nanosilica and supported the RTV silicon rubber induced by the nanostructure of the nanosilica. The research findings also point towards a highly durable superhydrophobic surface with good self-cleaning ability [16,36].

**Figure 8:**
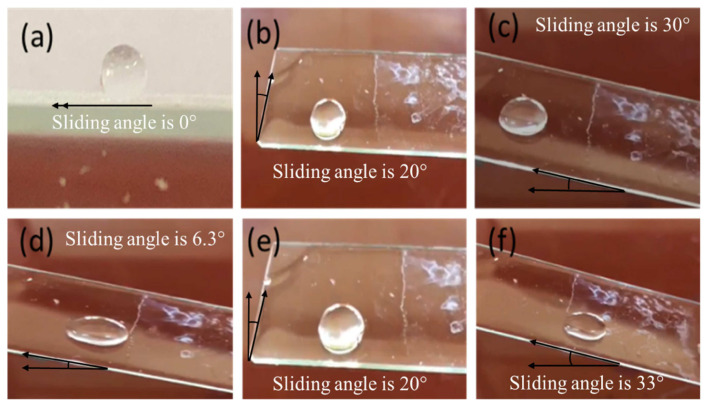
(a–f) the droplet sliding onto the superhydrophobic surface. The sliding angle is 6.3°.

A mixture of dust-water is supplied to study the sliding behavior of the droplet on a dry high voltage glass insulator. Figures 8a–8f show this process. When placing the droplet upon the glass sample, the droplet currently spreads like a pie cake due to the low surface tension on the glass surface using different inclination angles such as 33°, 30°, 20°, 6.3°, and 0°. Gravity, then, causes the water droplet to fall quickly. The excellent self-cleaning phenomenon of the nanocomposite superhydrophobic surface is indicated by the excellent sliding speed performance.

### 4.2. Dielectric strength

**Figure 9:**
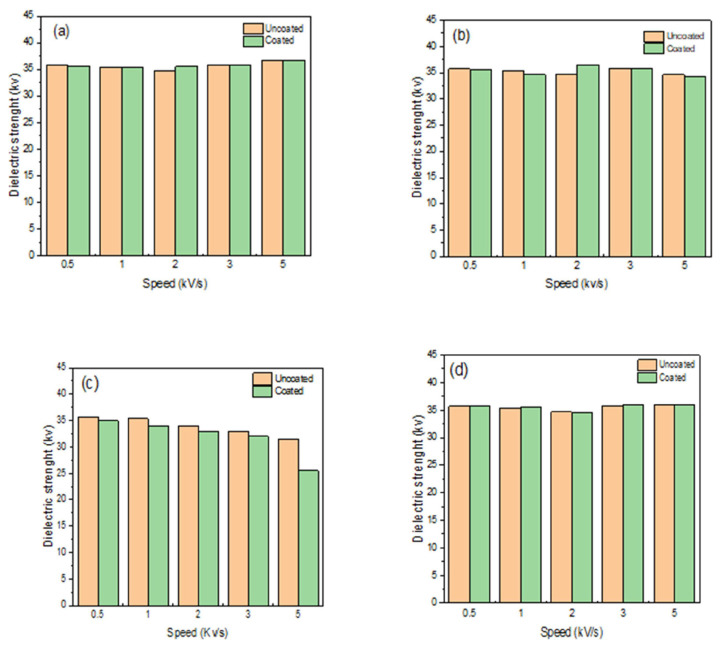
The dielectric breakdown-speed curve of a) the RTV silicon rubber/0 wt% nanosilica_,_ b) the RTV silicon rubber/23 wt% nanosilica, c) the RTV silicon rubber/33 wt% nanosilica, d) the RTV silicon rubber/44 wt% nanosilica.

The dielectric strength is the ability to withstand the electrical voltage up to the point of insulation breakdown and becoming a conductor. The dielectric strength analysis was conducted with different nanosilica concentrations such as 0 wt% nano-SiO_2_/RTV silicon rubber, 23 wt% nano-SiO_2_/RTV silicon rubber, 33 wt% nano-SiO_2_/RTV silicon rubber, 44 wt% nano-SiO_2_/RTV silicon rubber at speeds voltage of 0.5 to 5 kV/s. From Figure 9a, it was noticed that the dielectric strength value of the coated sample with 0 wt% nanosilica particles was 36.7 kV using a voltage speed of 5 kV/s. However, the dielectric strength was slightly reduced to 34.3 kV when using 23 wt% nano-SiO_2_/RTV silicon rubber as illustrated in Figure 9b. The dielectric strength was significantly changed to 25.5 kV using 33 wt% nano-SiO_2_/RTV silicon rubber, as shown in Figure 9c, improving the dielectric strength phenomenon. Nevertheless, the dielectric strength was increased to 36.0 kV using 44 wt% nano-SiO_2_/RTV silicon rubber. This result shows that a high percentage of the nanosilica particles (44 wt%) are converted to the heterojunction nanocomposite material as a semiconductor, confirming the unsuitability of the coating layers and the pre-collapse with failure observed. Furthermore, it shows that the speed voltage for the shedding purpose of the accelerating electrons could ionize the atoms and increase the ions’ numbers, thus, accelerating from the insulation to be a failure insulator [37].

### 4.3. Surface roughness

**Figure 10:**
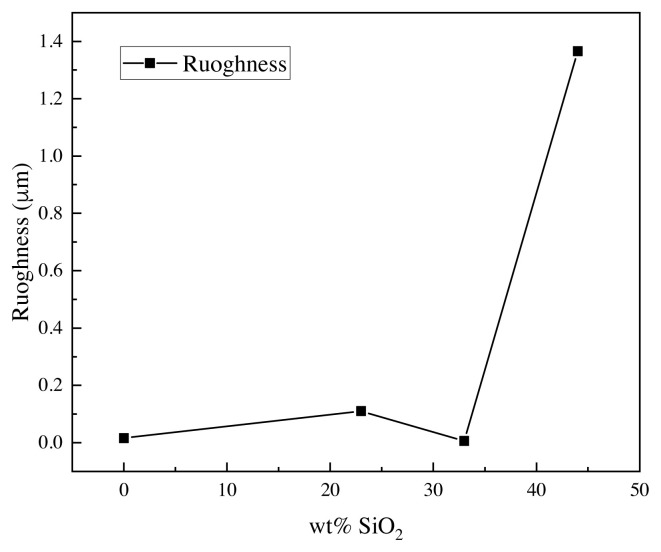
The surface roughness using different concentrations of the nanosilica with RTV silicon rubber.

The roughness test was conducted for the prepared samples such as the RTV silicon rubber/0 wt% nanosilica, RTV silicon rubber/23 wt% nanosilica, RTV silicon rubber/33 wt% nanosilica, and RTV silicon rubber/44 wt% nanosilica as illustrated in Figure 10. In Figure 10, the coating sample with 0 wt% nanosilica was examined and the roughness value was 0.016 μm due to the fact that no polymer matrix of the silicon rubber was used. The coated sample was tested using 23 wt% nanosilica and the roughness value was increased significantly to 0.11 μm, which is higher than when using 0 wt% nanosilica. Two different hypotheses have been offered to explain this phenomenon. On the one hand, the roughness increases the solid’s surface area, supporting hydrophobic geometrically (Wenzel model). However, because the drop sits partially on air, air might become trapped beneath it, resulting in a superhydrophobic behavior (Cassie model) [38]. The 33 wt% nanosilica particles were analyzed, and the roughness value was reduced to 0.006 μm, while when measuring the coated sample with a concentration of 44 wt% nanosilica, the roughness value was highly increased to 1.365 μm. This behavior is evidence that increase the nanosilica in the heterojunction nanocomposite RTV silicon rubber incorporated could increase the roughness value and then increase the contact angle between the water droplets and the glass surface. It is obvious that the distribution of nanoparticles using 33 wt% nanosilica results a very smooth surface due to the well-distribution of nano-silica particles on the glass surface, whereas the distribution of nanoparticles using 23 wt% nanosilica results in a relatively smooth surface due to the noncontinuation of the spread of nano-silica particles. The high concentration of the nanosilica could result in a cavitation with a high porosity, which would result in the presence of high roughness on its surface. Thus, the 33 wt% nanosilica was supported by the RTV silicon rubber and considered as the best value to reduce the roughness of the surface.

### 4.4. The flashover

**Figure 11:**
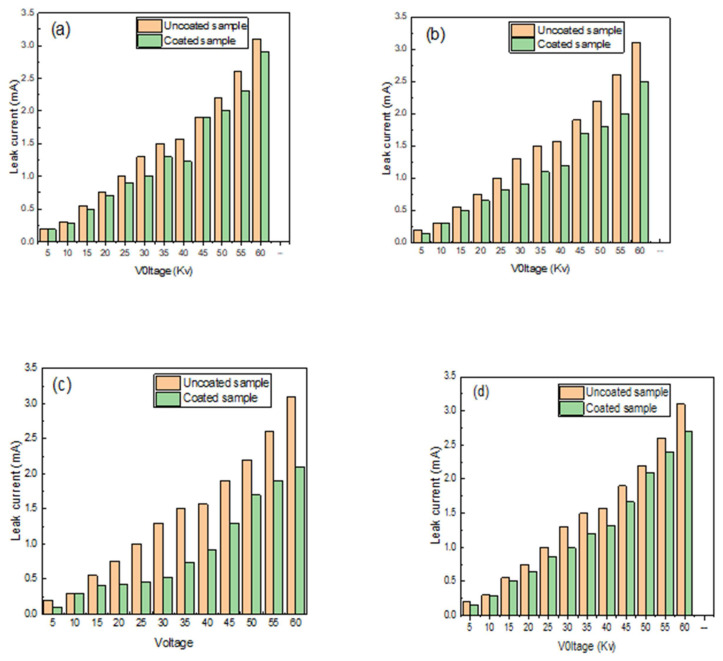
The flash over breakdown test using a wide range from the voltage (5 kV to 60 kV). (a) Pure RTV silicon rubber. (b) The RTV silicon rubber/23 wt% nanosilica. (c) The RTV silicon rubber/33 wt% nanosilica. (d) The RTV silicon rubber/44 wt% nanosilica.

The flashover, or current break down, is a condition of instantaneous flashing that occurs on power transmission lines of high and ultrahigh voltage due to contamination of glass insulators, adhesives, and natural pollutants such as dust or industrial dust materials, which come from the fumes of cement factories. As a result, the glass insulator could act as a semiconductor, sensing these power lines and turning them off and out of service. The flashover test was studied as illustrated in Figure 11. Different coatings on the high voltage glass insulator were investigated. According to Figure 11a, the leak current for an uncoated sample was 2.55 mA with a voltage load of 60 kV, whereas the leak current for the coated sample with pure RTV silicon was 2.25 mA, indicating that the coating slightly reduced the leak current. Also, the leak currents were reduced when using different voltage loads from 5 to 60 kV. This is attributed to the increased surface charge buildup when the uncoated surface retains larger drops of water containing pollutants that lead to an increased leakage current until the failure and the flashover breakdown occur [39].

From Figure 11b, Figure 11c, the leak currents were 3.2 and 2.5 mA using a voltage load of 60 kV; it is noticed that the leak current was significantly reduced using the heterojunction nanocomposite of the RTV/23 wt% nanosilica and the RTV/33 wt% nanosilica, respectively. However, using the RTV/44 wt% nanosilica (see Figure 11d), the leak current was increased to 2.54 kV, indicating that agglomeration of the nanosilica in the polymer matrix may have a negative effect on the flash efficiency of the heterojunction nanocomposite coating material. The evidence from this result suggests that the 33 wt% of the nanosilica immobilized in the nanocomposite structure is considered a perfect nano-layer that could cover the high voltage glass insulator perfectly, preventing the flashover dilemma.

## 5. The adhesion mechanism and the durability

**Figure 12:**
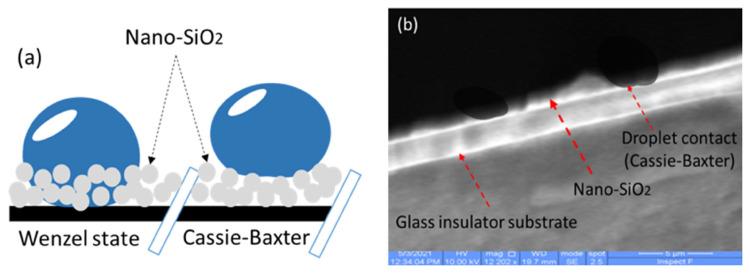
(a) Schematic illustration of the Wenzel & Cassie–Baxter phenomena, (b) FESEM photo (5 μm) to show the droplet contact with the coated nanocomposite surface with RTV silicon rubber/33 wt% nano-SiO_2_.

The wettability mechanism of the superhydrophobic surface is fundamentally divided into two states: The Wenzel state, which reflects high adhesion, and the Cassie–Baxter state, which reflects low adhesion on the surface. Figure 12a illustrates the difference between the Wenzel state and the Cassie–Baxter state. When droplets cling to the surface, it indicates that high adhesion was achieved via the Wenzel state phenomenon of the hydrophobic surface. The superhydrophobic surface is reached by air, as a cushion is entrapped through the porous solid surface, which is called the Cassie–Baxter phenomenon. Consequently, spherical water drops are comfortably spilled off the coated nanocomposite RTV silicon rubber/33 wt% nanosilica onto the high voltage glass insulator with a contact angle of 158° ± 3 as displayed in the FESEM photo Figure 12b. Therefore, the micro-coated structure composed of the nanomorphology on the surface of the glass insulator was reported to be in charge of a low rolling angle with the glass insulator [19,40]. The current findings add substantially to our understanding that the droplet was suspended above the coated nanocomposite superhydrophobic surface, which is ascribed to the Cassie phenomenon.

To investigate the real durability of the coated sample with the nanocomposite of the RTV/33 wt% nanosilica under ambient conditions, the sample was placed in the outside region for 30 days, and the contact angle was measured to be above 158°±6, demonstrating the superhydrophobicity was conserved on its surface. However, a slight decrease in the contact angle was noticed after 15 days to 154.6° ± 4 due to the increased humidity at night. On the twentieth day, after the sample was exposed to sunlight for 1 day, the contact angle of the sample was again significantly increased and reached 156°±3. Therefore, the nanocomposite coating material has a high robustness and can be used for a long time in various ecosystems.

## 6. The application in the real environment

**Figure 13:**
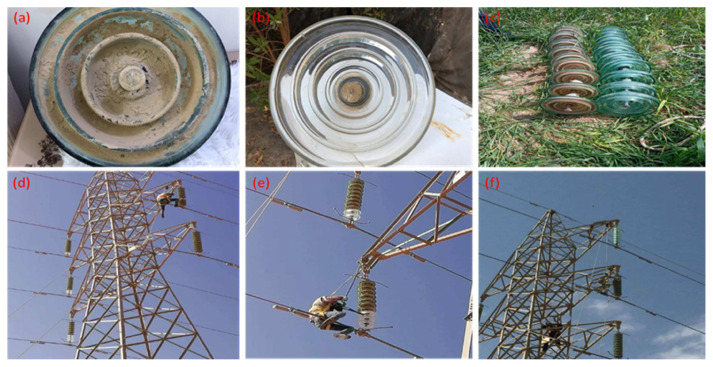
(a) dirty high voltage glass insulator, (b) it’s cleaned up and then coated by nanocomposite material, (c) a set of the coated and uncoated high voltage glass insulator, (d–f) the installation process of the high voltage glass insulator.

To apply the coated glass insulator environmentally, a series of ten insulators as coated and uncoated discs for one of the electric power transmission lines with a voltage of 132 kV between the two stations of the North Karbala-Al-Ukhaidir station (192 km south-west of Baghdad, Iraq) were applied. Two path lines, each of which was 88 kilometers long, were tested. These lines were chosen in this work due to their being exposed to the harshest conditions of environmental pollution from the product dust of the industrial pollution field, since the path of these lines passes through spaces with fumes and dirt residues from cement factories, thermostone, and gypsum dust. It was observed that the installation during a period of 3–5 months showed a clear difference between the coated sample and the uncoated insulator discs in terms of the adhesion of dust and residues. As shown in Figure 13a–13f, it was also discovered that when a whole series of coated insulators were connected to the transmission line of one phase (R), and uncoated glass insulators were connected to the transmission lines of the other phases (S, T); the majority of the separation cases occurred for the transmission lines referring to one of the two phases (S or T) with no recording sensitivity signs for the R phase. This indicates that the coated surface has a great role in the dielectric strength with no reduction in efficiency; additionally, it obtains the lowest value of current leakage at 2.5 mA with no flashover state. This real application gave evidence of the durability of the coated glass insulator and its longevity, with no loss in efficiency over a long period of time.

## 7. Conclusion

The purpose of the current study was to design a novel nanocomposite superhydrophobic nano-SiO_2_ particles incorporated into RTV silicon rubber by the modified sol-gel technique coating a high voltage glass insulator and evaluate the resistance against dusty weather. This study has shown that the RTV silicon rubber immobilized 33 wt% of the prepared nano-SiO_2_ increased the contact angle to a surprising 158° ± 3 obtaining an almost completely smooth surface with no adhesive of the water-dust droplets. One of the more significant findings to emerge from this study is that the flashover is extremely reduced to 2.5 mA with a loading voltage of 60 kV. It was also shown that the compatibility between the prepared nano-SiO_2_ and the RTV silicon rubber significantly boosted the high adhesive on the glass insulator. The third major finding was that a high uniform roughness surface was obtained with a 0.006 μm using 33 wt% nano-SiO_2_ in the nanocomposite of RTV silicon rubber, preserving its durability with a uniform thickness of 1μm. The current study makes significant contributions to self-cleaning applications, increasing the power transmitted over long distances without requiring service interruption due to rain-dust storms.

## Supplementary Material

Figure S1 Performance of the high-voltage glass insulator flashover subjected to fast transient overvoltages test

Figure S2 Water contact angles on the natural glass (no coating), glass coating with RTV silicon rubber & RTV silicon rubber/33% nanosilica.

Figure S3 Dust-water contact angles on the natural glass (no coating), glass coating with RTV silicon rubber & RTV silicon rubber/33% nanosilica.

Figure S4 High-voltage glass insulator (a) No coating (pristine glass insulator (b) Coating with RTV silicon rubber/33% nanosilica
