# Supplementary figures and images for: Synthesis of a novel antiweathering nanocomposite superhydrophobic room temperature vulcanized (RTV) silicon rubber enhanced with nanosilica for coating high voltage insulators
Source: Turk J Chem. 2021 Dec 27;46(3):704–20. doi: 10.55730/1300-0527.3361 (PMC10503989; doi:10.55730/1300-0527.3361)

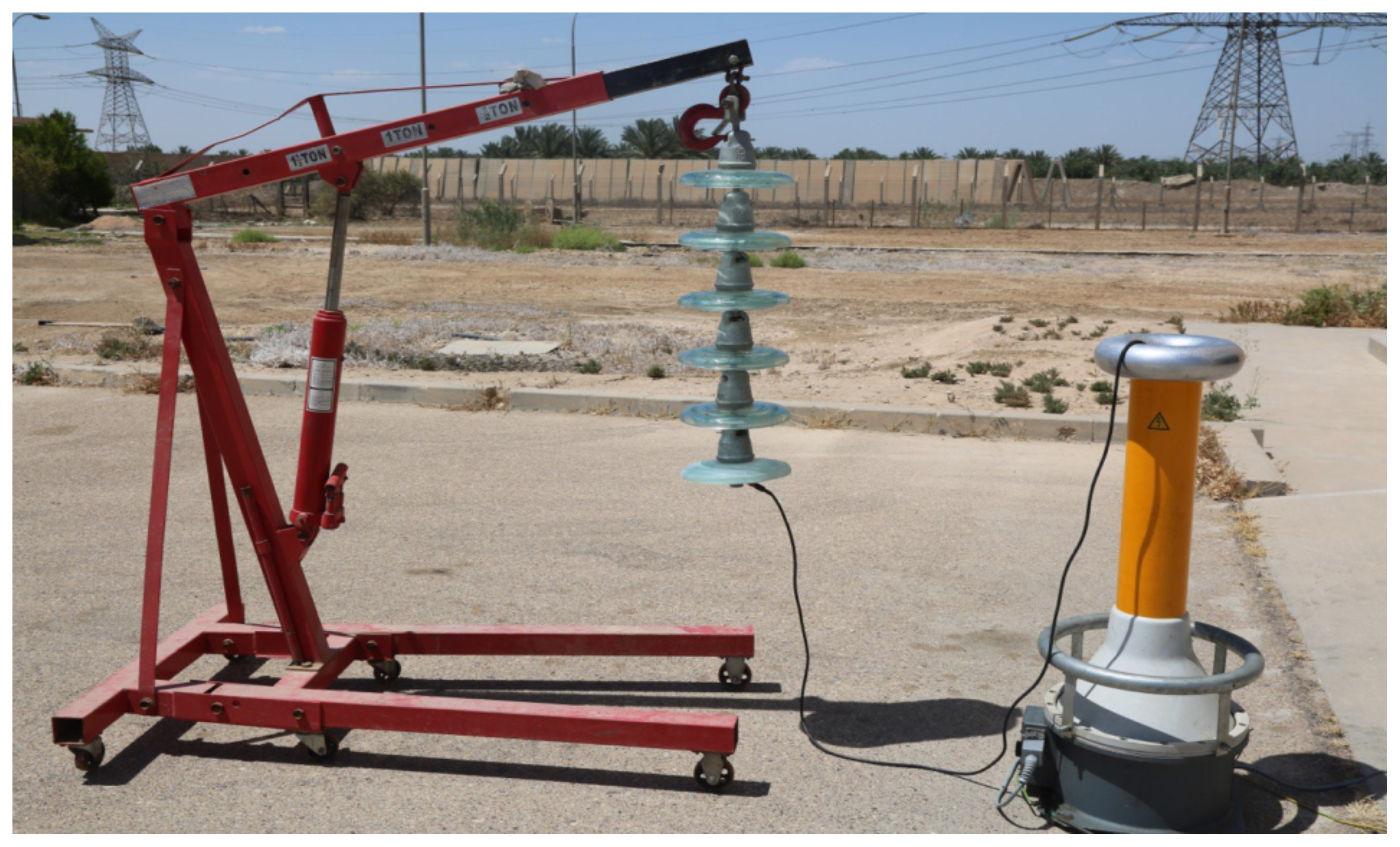

Supplement: Figure S1 — Performance of the high-voltage glass insulator flashover subjected to fast transient overvoltages test [file turkjchem-46-3-704s1.tif]

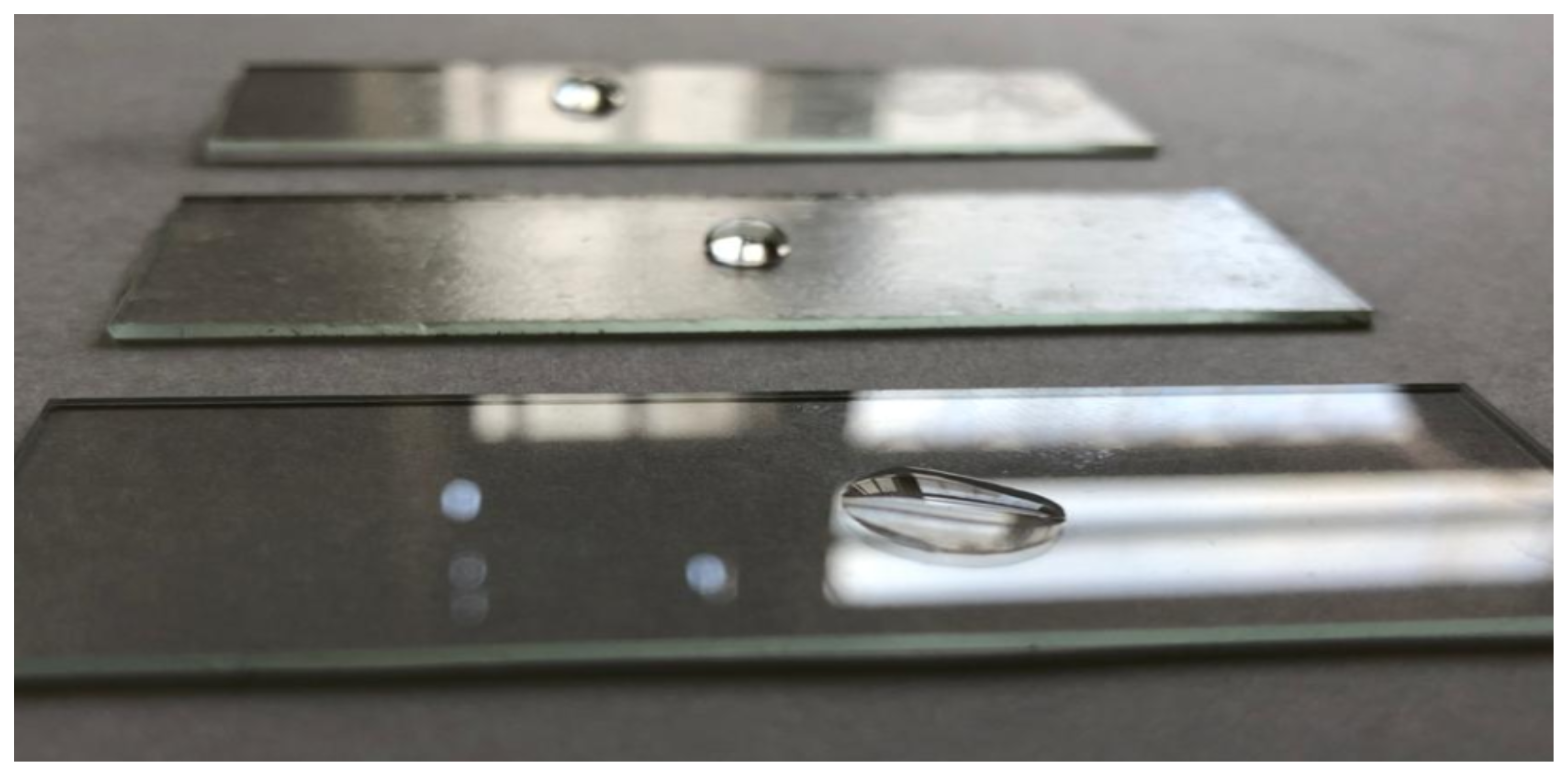

Supplement: Figure S2 — Water contact angles on the natural glass (no coating), glass coating with RTV silicon rubber & RTV silicon rubber/33% nanosilica. [file turkjchem-46-3-704s2.tif]

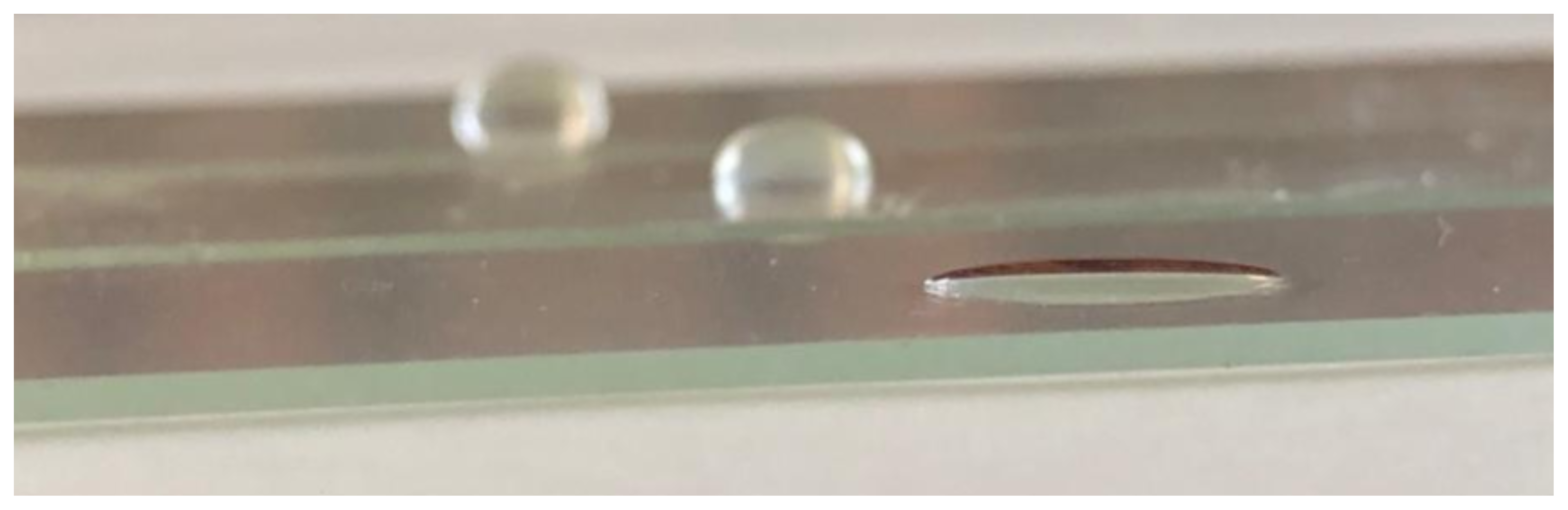

Supplement: Figure S3 — Dust-water contact angles on the natural glass (no coating), glass coating with RTV silicon rubber & RTV silicon rubber/33% nanosilica. [file turkjchem-46-3-704s3.tif]

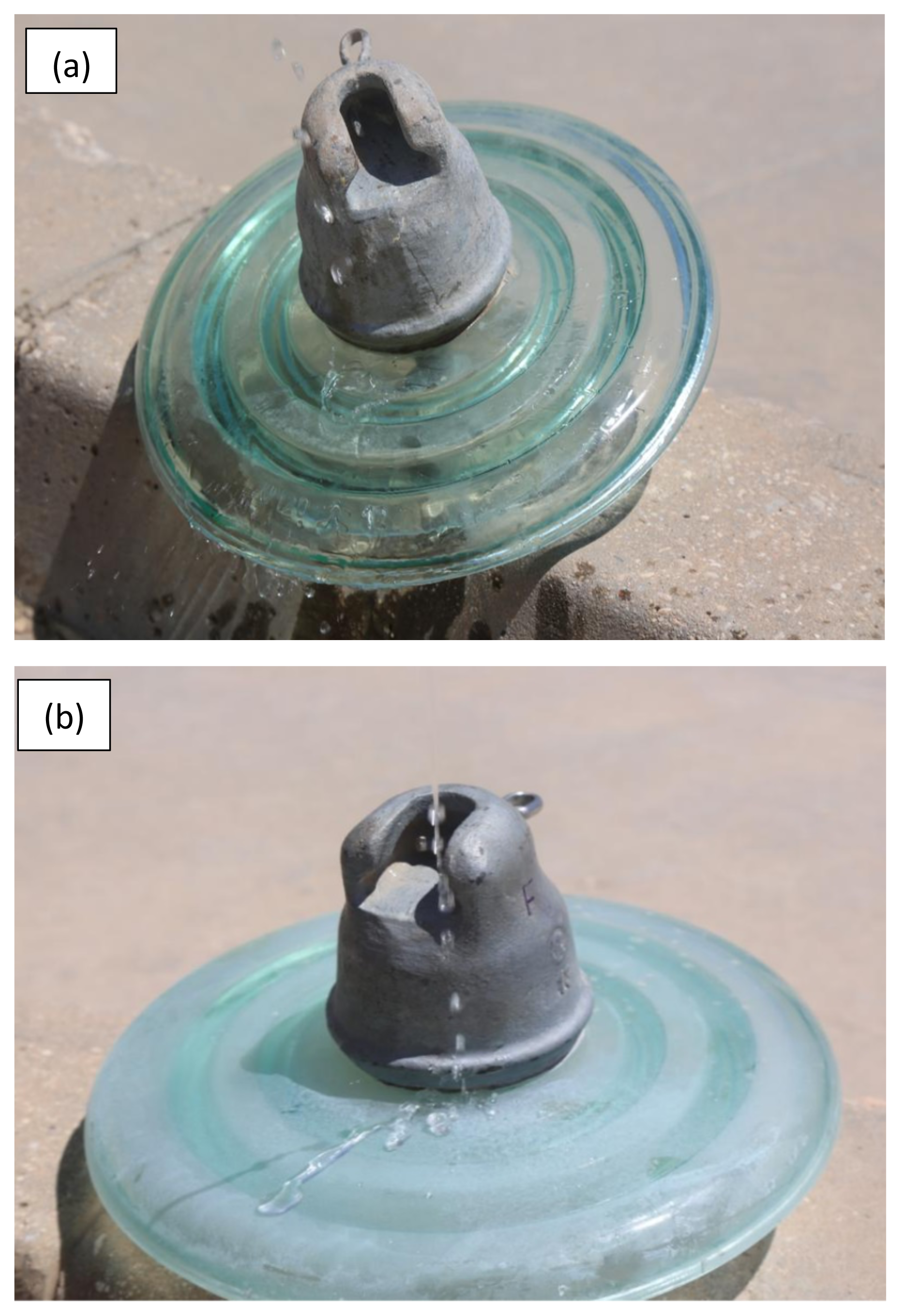

Supplement: Figure S4 — High-voltage glass insulator (a) No coating (pristine glass insulator (b) Coating with RTV silicon rubber/33% nanosilica [file turkjchem-46-3-704s4.tif]
